# High Throughput Virtual Screening and Molecular Dynamics Simulation for Identifying a Putative Inhibitor of Bacterial CTX-M-15

**DOI:** 10.3390/antibiotics10050474

**Published:** 2021-04-21

**Authors:** Shazi Shakil, Syed M. Danish Rizvi, Nigel H. Greig

**Affiliations:** 1 King Fahd Medical Research Center, King Abdulaziz University, Jeddah 21589, Saudi Arabia; 2 Department of Medical Laboratory Technology, Faculty of Applied Medical Sciences, King Abdulaziz University, Jeddah 21589, Saudi Arabia; 3 Center of Excellence in Genomic Medicine Research, King Abdulaziz University, Jeddah 21589, Saudi Arabia; 4 Department of Pharmaceutics, College of Pharmacy, University of Hail, Hail 81481, Saudi Arabia; sm.danish@uoh.edu.sa; 5 Translational Gerontology Branch, Intramural Research Program, National Institute on Aging, National Institutes of Health, Baltimore, MD 21224, USA; greign@grc.nia.nih.gov

**Keywords:** antibiotic resistance, CTX-M-15, docking, extended spectrum β-lactamase, molecular dynamics simulation, screening

## Abstract

Background: Multidrug resistant bacteria are a major therapeutic challenge. CTX-M-type enzymes are an important group of class A extended-spectrum β-lactamases (ESBLs). ESBLs are the enzymes that arm bacterial pathogens with drug resistance to an array of antibiotics, notably the advanced-generation cephalosporins. The current need for an effective CTX-M-inhibitor is high. Objective: The aim of the current study was to identify a promising anti-CTX-M-15 ligand whose chemical skeleton could be used as a ‘seed-molecule’ for future drug design against resistant bacteria. Methods: Virtual screening of 5,000,000 test molecules was performed by ‘MCULE Drug Discovery Platform’. ‘ADME analyses’ was performed by ‘SWISS ADME’. TOXICITY CHECKER of MCULE was employed to predict the safety profile of the test molecules. The complex of the ‘Top inhibitor’ with the ‘bacterial CTX-M-15 enzyme’ was subjected to 102.25 ns molecular dynamics simulation. This simulation was run for 3 days on a HP ZR30w workstation. Trajectory analyses were performed by employing the macro ‘md_analyze.mcr’ of YASARA STRUCTURE version 20.12.24.W.64 using AMBER14 force field. YANACONDA macro language was used for complex tasks. Figures, including RMSD and RMSF plots, were generated. Snapshots were acquired after every 250 ps. Finally, two short videos of ‘41 s’ and ‘1 min and 22 s’ duration were recorded. Results: 5-Amino-1-(2H-[1,2,4]triazino[5,6-b]indol-3-yl)-1H-pyrazole-4-carbonitrile, denoted by the MCULE-1352214421-0-56, displayed the most efficient binding with bacterial CTX-M-15 enzyme. This screened molecule significantly interacted with CTX-M-15 via 13 amino acid residues. Notably, nine amino acid residues were found common to avibactam binding (the reference ligand). Trajectory analysis yielded 410 snapshots. The RMSD plot revealed that around 26 ns, equilibrium was achieved and, thereafter, the complex remained reasonably stable. After a duration of 26 ns and onwards until 102.25 ns, the backbone RMSD fluctuations were found to be confined within a range of 0.8–1.4 Å. Conclusion: 5-Amino-1-(2H-[1,2,4]triazino[5,6-b]indol-3-yl)-1H-pyrazole-4-carbonitrile could emerge as a promising seed molecule for CTX-M-15-inhibitor design. It satisfied ADMET features and displayed encouraging ‘simulation results’. Advanced plots obtained by trajectory analyses predicted the stability of the proposed protein-ligand complex. ‘Hands on’ wet laboratory validation is warranted.

## 1. Introduction

Multidrug resistant bacteria are a major therapeutic challenge [1,2,3]. Pathogenic Gram negative bacteria are considered to be among the greatest problem-causing entities by The Infectious Diseases Society of America [4]. The generation of β-lactamases is the leading cause of resistance to β-lactam antibiotics in Gram-negative bacteria [5]. Two schemes are noteworthy for classification of beta-lactamases. These are the Ambler molecular classification and the updated Bush–Jacoby functional classification [6,7,8]. The Ambler scheme classifies beta-lactamases into four classes according to the protein homology. Beta-lactamases of class A, C, and D are serine β-lactamases, and class B enzymes are metallo-beta-lactamases [6]. The updated Bush–Jacoby–functional scheme is based on substrate and inhibitor profiles so as to group the enzymes in a manner that could be correlated with their phenotype in clinical isolates [8].

CTX-M-15 is a Class A beta lactamase. It is placed in group 2be of the functional classification [8]. These enzymes cleave the amide bond present in the β-lactam ring and, thereby, make β-lactam antibiotics harmless to bacteria [9]. The alteration in activities of CTX-M enzyme that may lead to the evolution of more variants may be due to point mutations present either inside or outside of the active site omega loop (amino acid positions 161 to 179). We had described the interaction of CTX-M-15 with cefotaxime prior to the availability of any crystallization data [10]. It is an important type of extended spectrum β-lactamases (ESBLs). ESBLs are the enzymes that provide bacterial pathogens with drug resistance to an array of antibiotics; notably, the advanced-generation cephalosporins [11,12]. CTX-M is among the most prevalent ESBLs exhibiting global dissemination, while CTX-M-15 has been found to be the most prevalent variant by an array of studies [13,14].

The discovery and design of novel β-lactamase inhibitors to thwart the ever looming threat of multidrug resistant bacteria remains a hot topic among clinicians, microbiologists, and drug discovery scientists [3,11]. Carbonitrile derivatives have been reported to display antimicrobial activities against resistant bacterial strains by many authors [15,16,17]. In a study, authors reported the synthesis of new carbonitrile derivatives as potential antimicrobials [18]. In silico screening coupled to molecular simulations has become an increasingly indispensable part of current drug discovery protocols [19,20,21]. The present need for an effective CTX-M-inhibitor is high. The aim of the current study was to identify a promising anti-CTX-M-15 ligand whose chemical skeleton could be used as a ‘seed-molecule’ for future drug design against resistant bacteria. Relevant computational approaches that included screening, docking, ADMET analyses, and a detailed 102.25 ns molecular dynamics simulation were employed to achieve the objective. Accordingly, the present study reports a carbonitrile-based anti-CTX-M-15 ligand.

## 2. Results and Discussion

### 2.1. CASTp3.0 Protein Topography Probe Result

Examination of the complexed crystal (PDB ID: 4HBU; Resolution: 1.10 Å) focused on the avibactam binding amino acid residues of the bacterial CTX-M-15 protein employing the default probe radius of 1.4Å in CASTp3.0, revealed that this interaction mainly involved 14 residues [22]. These crucial residues were Cys 69, Ser 70, Lys 73, Asn 104, Tyr 105, Ser 130, Asn 132, Asn 170, Thr 216, Lys 234, Thr 235, Gly 236, Ser 237, and Gly 238.

### 2.2. Outcome of the Screening Funnel

**Figure 1:**
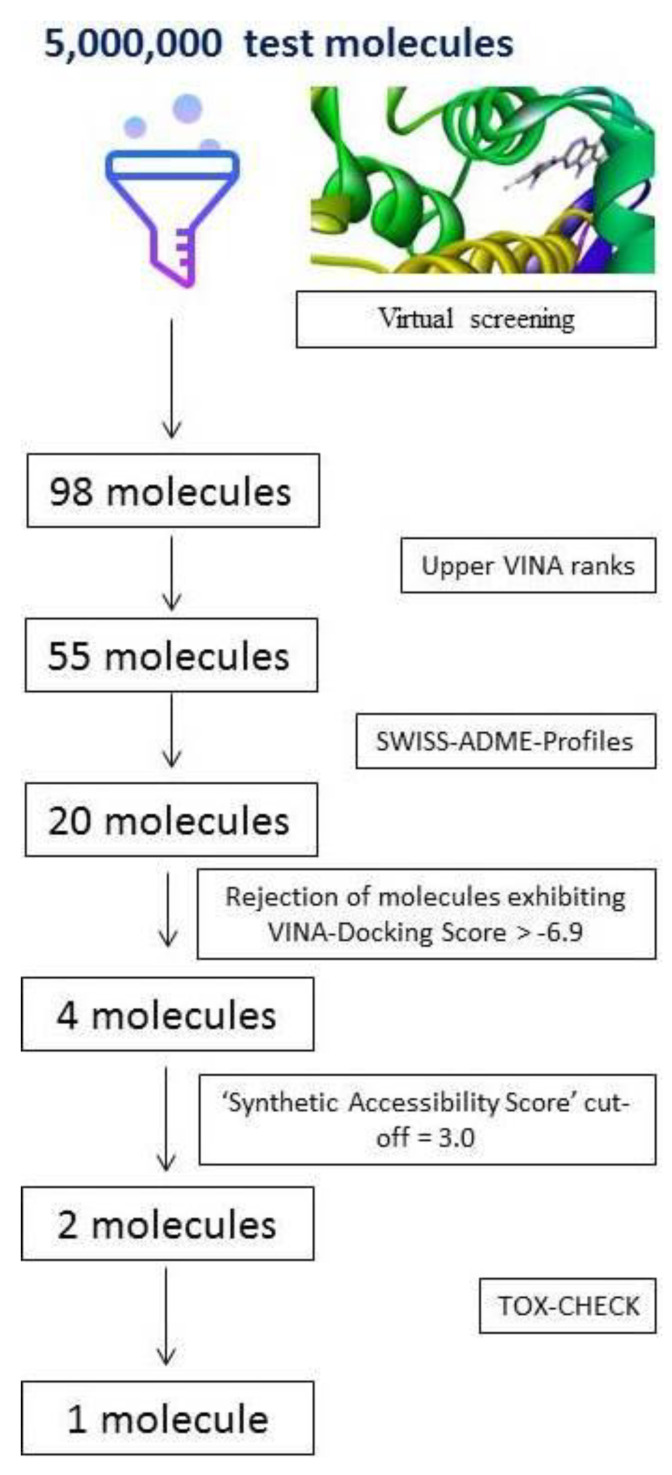
The screening funnel for 5,000,000 test molecules targeting inhibition of bacterial CTX-M-15 protein.

Structure-based in silico screening is generally concerned with identifying a previously unconsidered series of ligands that are predicted to inhibit a binding site on a protein of interest, and are found by rigorous scanning of vast ‘small molecule databases’. In the present study, a screening funnel for 5,000,000 test molecules against bacterial CTX-M-15 enzyme, led to a set of 98 putative inhibitors; Figure 1.

### 2.3. VINA Ranks and ADME-Analyses Results

It is worth mentioning that the authors had previously published the “CLICK BY CLICK” protocol [19] for non-bioinformaticians to support their ability to work with AUTODOCK [23], and incorporate it into their research. In the present article, we have used AutoDock Vina for screening (implemented within the screening platform). VINA is known to significantly improve the average accuracy of the binding mode predictions compared to AutoDock 4 [24]. Accordingly, VINA has created its own space among several important software packages used to screen vast databases that comprise of millions of candidate ligands [24]. Top VINA scores are generally regarded as indicators of more efficient binding interactions. In this light, 55 molecules possessing the upper VINA scores were chosen for further filtration by SWISS ADME [25] (Figure 1). Table 1 shows the SWISS ADME profiles of the top four inhibitors obtained from the screening of 5 million candidate molecules against CTX-M-15 enzyme Table 1.

Ligand molecules that display poor actual or predicted pharmacokinetic profiles tend to be rejected during the initial stages of the drug-design process. For imparting early flexibility into the screening process, we allowed retention of all ligands that passed at least four out of the six filters detailed in Table 1, namely Lipinski (Pfizer), Ghose, Veber (GSK), Egan (Pharmacia), Muegge (Bayer), and PAINS [16,17,18,19,20]. Furthermore, we permitted one RO5 violation for the same reason, to support search broadness and flexibility. Specifically, Lipinski’s RO5 to predict orally-active drug molecules for use in humans, suggests that promising agents should possess a molecular mass < 500 Da, a log P-value of not more than 5, no more than five H-bond donors, no more than 10 Hydrogen-bond acceptors, and a molar of refractivity of between 40–130 [26]. Accordingly, 20 molecules (out of 55) were selected.

### 2.4. Outcome of Docking Score, ‘Zero RO5 Violation’, ‘Synthetic Accessibility Score’ and TOX-CHECK

**Figure 2:**
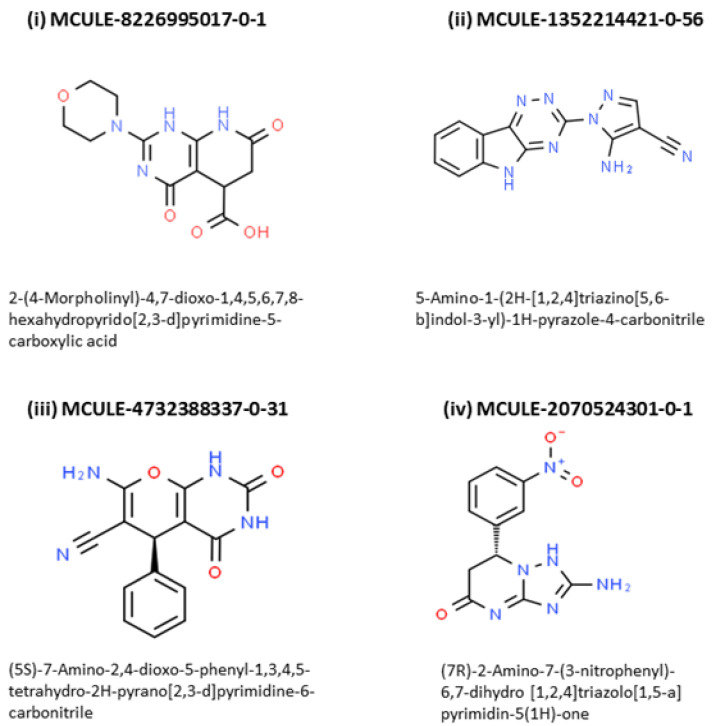
Chemical structures of the four upper ranking inhibitors of the screening study.

With reference to the screening platform used herein, ‘VINA-docking scores’ were assigned to each of the test ligands. The ranking process could be understood from the following example. A ligand displaying a VINA-docking score of −7.2 would be ranked above the ligand displaying a docking score of −7.0. The upper layer ligands might be expected to have overall efficient binding interactions with the corresponding protein. The detailed methodology and formulae involved in the calculation of the VINA-scoring function can be referred from the highly cited article [24]. Inhibitors, whose dissociation rate with the binding target is lower, might be expected to possess an increased half-life [27]. Molecules exhibiting VINA-docking scores > −6.9 and/or violating 1 or more ‘RO5’ were rejected. The choice of this value (−6.9) was totally arbitrary. It was done simply to exclude the lower ranking ligands and narrow down the number for further filtration steps. We were left with four putative inhibitors. These four ligands were identified as: MCULE-8226995017-0-1, MCULE-1352214421-0-56, MCULE-4732388337-0-31, and MCULE-2070524301-0-1 in the database. Two of the four ligands were found to possess a ‘Synthetic Accessibility Score’ of greater than 3.0, and hence were rejected. Candidate molecules possessing moieties/sub-structures that might be regarded as signatures or hallmarks of known toxic entities are generally rejected during the screening process. Accordingly, only one of the two remaining ligands, namely MCULE-1352214421-0-56 proved able to pass the ‘Toxicity Checker’ filter. The ‘SMILES notation’ of this top putative inhibitor was entered into CHEMSPIDER to generate the corresponding IUPAC name; specifically, 5-Amino-1-(2H-[1,2,4]triazino[5,6-b]indol-3-yl)-1H-pyrazole-4-carbonitrile (Figure 2, compound (**ii**)). Molecule ‘Leads’ are normally expected to possess a XLogP3 within 3.5. In this regard, MCULE-1352214421-0-56 displayed a XLogP3 value as 1.31. The ligand hence displayed a high predicted gastrointestinal absorption, which is considered as a good indicator concerning putative drugs for oral administration. The ligand was additionally predicted to be CNS-inactive in relation to its projected ability to cross the blood-brain barrier, a valuable feature to limit off-target effects. In the field of drug design, as a generalization, molecules possessing lower numbers of rotatable bonds are preferred. MCULE-1352214421-0-56 possesses a single rotatable bond. Accordingly, MCULE-1352214421-0-56 (5-Amino-1-(2H-[1,2,4]triazino[5,6-b]indol-3-yl)-1H-pyrazole-4-carbonitrile) was designated as the ‘Top putative CTX-M-15 inhibitor’ in the present screening study. In synopsis, it displayed zero RO5 violations and generously passed all the major drug-screen filters, as well as the ‘toxicity checker’.

### 2.5. Molecular Overlay and Binding Interactions

The chemical structures of the four upper ranking inhibitors of the screening study are presented in Figure 2.

**Figure 3:**
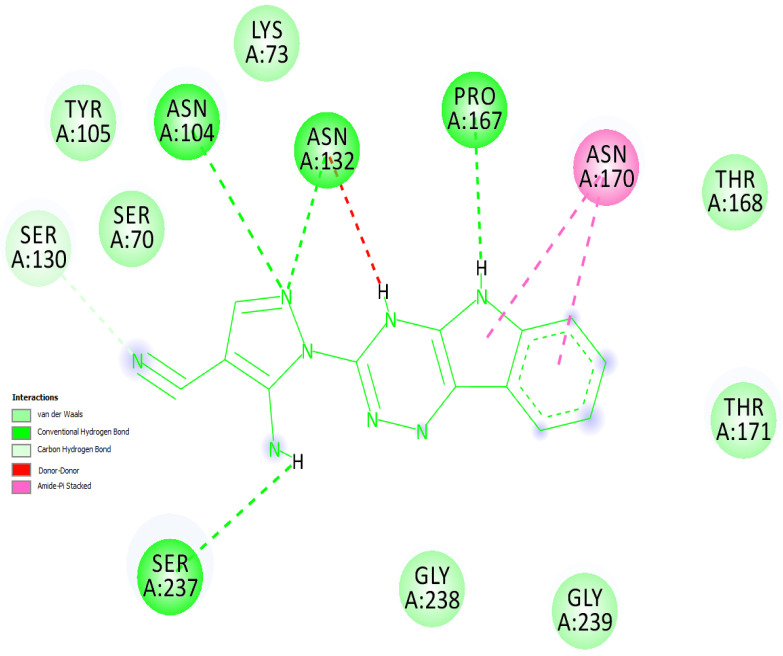
‘2-D-Diagram’ of binding interactions for the complex between the top inhibitor and bacterial CTX-M-15.

The docking results (notably the docked complex of the top screened out inhibitor, MCULE-1352214421-0-56 with the bacterial CTX-M-15 protein) were re-confirmed by two other platforms, namely “YASARA GLOBAL DOCKING” (included within licensed YASARA STRUCTURE) and licensed “DOCKINGSERVER” [Virtua Drug Ltd., Budapest, Hungary]. It is imperative to mention that the results by the aforementioned platforms were found to be in perfect harmony with each other for the aforementioned complex. Figure 3 shows the ‘2-D-Diagram’ of binding interactions for the complex between the top inhibitor and bacterial CTX-M-15. Interacting amino acid residues are marked and a ‘key’ to color codes representing various interactions is duly provided in Figure 3. The complete log file for ‘YASARA GLOBAL DOCKING-Result Analysis’ has been provided as Supplementary Material Supplementary Text S2.

**Figure 4:**
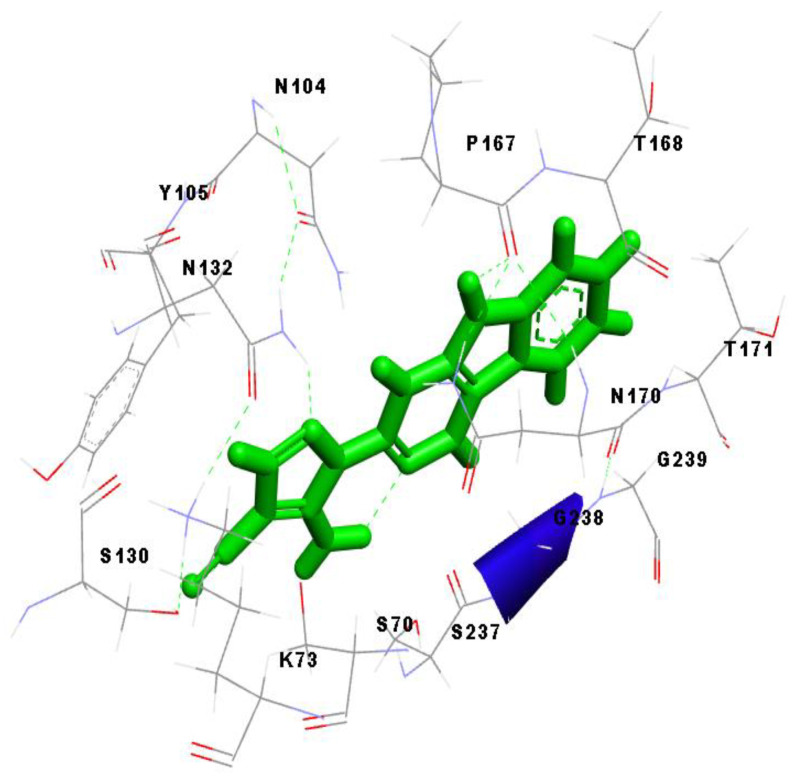
A three-dimensional representation of the binding site of CTX-M-15 protein interacting with the top ligand, ‘MCULE-1352214421-0-56’.

Furthermore, we compared the binding interactions of 5-Amino-1-(2H-[1,2,4]triazino[5,6-b]indol-3-yl)-1H-pyrazole-4-carbonitrile with that of avibactam with reference to their respective complexes with CTX-M-15. The former interacted with CTX-M-15 via 13 amino acid residues. It is significant that nine of these amino acid residues were found identical to that of the CTX-M-15-Avibactam complex. The common residues were identified as Asn104, Tyr105, Asn132, Asn170, Gly238, Ser237, Ser70, Lys73, and Ser130. It is worth mentioning that the binding pocket for the ‘reference ligand’ and the ‘top-inhibitor’ was confirmed to be the same; as also indicated by the nine residues that were found common to the aforementioned complexes. The readers are encouraged to refer to Section 2.1 of this manuscript at this point (for a clear understanding). Figure 4 exclusively shows the binding site of CTX-M-15 protein interacting with the top ligand, MCULE-1352214421-0-56 in a three-dimensional representation; Figure 4.

**Figure 5:**
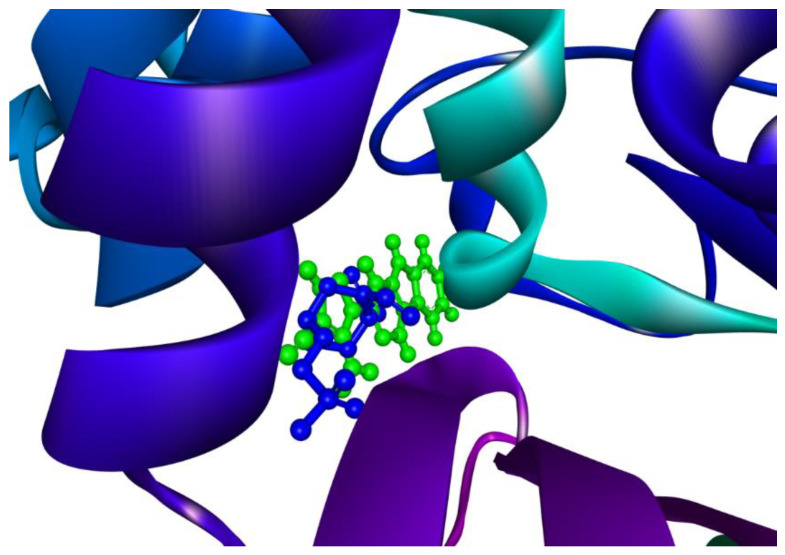
‘Molecular Overlay’ diagram of the ‘top inhibitor’ interacting with bacterial CTX-M-15 alongside ‘Avibactam’ (the reference inhibitor). The ‘top inhibitor’ and ‘Avibactam’ are represented as ‘ball and stick’ models in green and blue colors, respectively.

The ‘Molecular Overlay’ tool of Discovery Studio Visualizer was employed to generate another useful figure; Figure 5. It simultaneously displays Avibactam (reference ligand) as well as the ‘top molecule’ obtained in the current screening study interacting with the CTX-M-15 protein to provide a clear comparison of their respective alignments; Figure 5. 5-Amino-1-(2H-[1,2,4]triazino[5,6-b]indol-3-yl)-1H-pyrazole-4-carbonitrile and Avibactam are represented as ‘ball and stick’ models in green and blue colors, respectively; Figure 5.

### 2.6. Outcome of Molecular Dynamics Simulation (102.25 ns)

**Figure 6:**
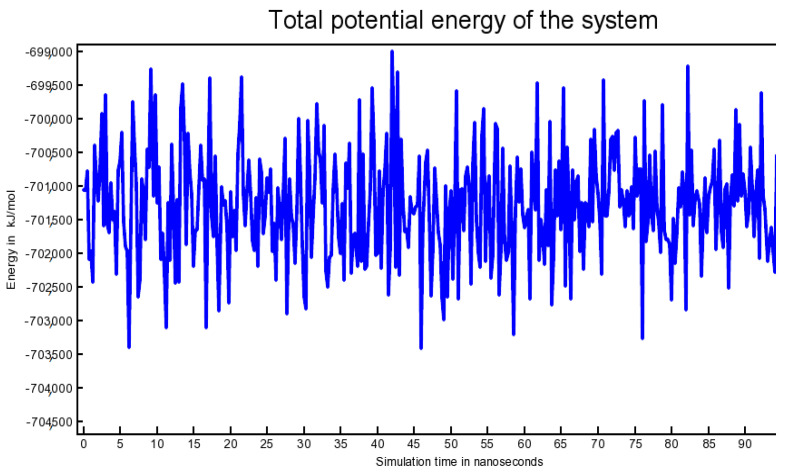
Total potential energy of the system plotted against simulation time.

A trajectory analysis by YASARA STRUCTURE version 20.12.24.W.64 over the duration of 102.25 ns time, using AMBER14 force field, yielded 410 snapshots. This simulation was run for 3 days on a HP ZR30w workstation. Video clips of ‘41 s’ and ‘1 min and 22 s’ were additionally captured. It is noteworthy that we repeated molecular dynamics simulation trials three times and each time the results were found to be in harmony with each other. The term ‘Solute’ used in the present article (in line with the analyses results provided by YASARA STRUCTURE) denotes the ‘Protein-ligand complex’. Figure S1 of the ‘Supplementary File’ provides a ray-traced picture of the protein ligand complex subjected to simulation; Figure S1. A separate ray-traced diagram for the ‘top CTX-M-15-inhibitor’ was, likewise, generated. Conformational alterations in the simulated protein (bacterial CTX-M-15) lead to density fluctuations. However, the molecules in the human body “live” in a constant cell pressure. To account for this, the ‘simulation cell’ was re-calibrated for maintaining a fixed pressure throughout the simulation. The total potential energy of the system was plotted against simulation time; Figure 6.

**Figure 7:**
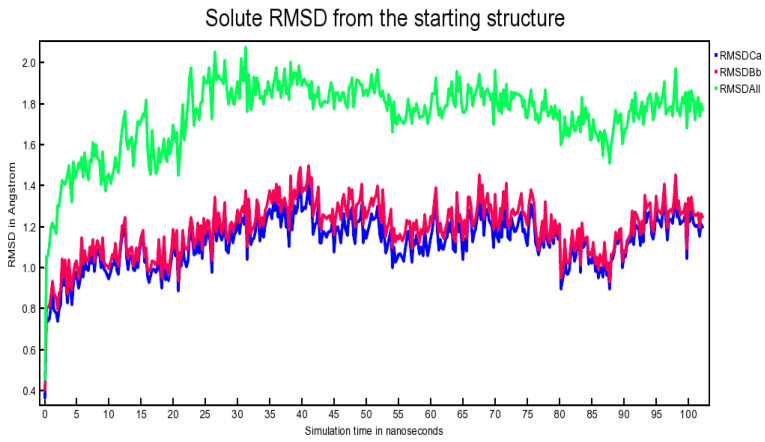
Solute RMSD from the starting structure plotted against the simulation time.

When the simulation is started from an energy-minimized “frozen” conformation, there is usually a sharp increase in energy during the first picoseconds, since the added kinetic energy is partly stored as potential energy. Also on a larger time-scale, the potential energy will often not decrease; a common reason being the counter ions. These are initially placed at the positions with the lowest potential energy, usually close to charged solute groups. From this place, they detach to gain entropy and also potential energy. It can be inferred from the above plot that during the progress of simulation, the total potential energy of the system fluctuated within an acceptable range of −703,500 kJ/mol to −699,000 kJ/mol Figure 6, thereby indicating the validity of the simulation. The Van der Waals, molecular, and solvent-accessible surface areas of the solute in Å^2^ were also determined against simulation time. Protein–ligand contacts against simulation time were plotted to aid understand how the binding patterns evolved as the simulation progressed. Hydrogen bonds, hydrophobic contacts, and ionic interactions were generated as red, green, and blue dots, respectively. Mixtures of these three colors indicate that a certain residue might have involvement in multiple kind of interactions with the ligand [data not shown for brevity]. Figure 6 shows the Calpha [RMSDCa], backbone [RMSDBb], and all-heavy atom [RMSDAll] RMSDs plotted against the simulation time. Figure 7 shows ‘solute RMSD from the starting structure’ plotted against the simulation time; Figure 7.

It can be inferred from the above plot that around 26 ns, equilibrium was achieved and, thereafter, the complex remained reasonably stable. After a duration of 26 ns and onwards until 102.25 ns, the backbone RMSD fluctuations were found to be confined within a range of 0.8–1.4 Å. This narrow range was considered tolerable for the aforementioned plot; Figure 7. The root mean square fluctuation (RMSF) per solute residue was calculated from the average RMSF of the atoms constituting the residue, and the RMSF plot was generated. An array of tables and advanced plots including the ‘dynamic cross-correlation matrix’ were, likewise, generated. Results from these additional plots, together, re-enforced the stability of the proposed complex [plots not shown for brevity].

Furthermore, we analyzed three pdb files for the complex of bacterial CTX-M-15 with 5-Amino-1-(2H-[1,2,4]triazino[5,6-b]indol-3-yl)-1H-pyrazole-4-carbonitrile using Discovery Studio Visualizer. These files were as follows: (i) the highest ranking docked complex obtained by YASARA GLOBAL DOCKING; (ii) snapshot with minimum solute energy, retrieved from results analysis report of simulation implemented in YASARA STRUCTURE; and (iii) the last snapshot of simulation retrieved from YASARA STRUCTURE, after the execution of the command contained in the macro, ‘md_analyze.mcr’. Not surprisingly, it was found that the binding pocket was essentially the same in all of these three files, as confirmed by the presence of the same crucial amino acid residues, which were known to constitute the binding crevice, example N104, P167, N170, G238, S237. This definitely confirmed that the ligand did not bind to any unintended groove during docking as well as during molecular dynamics simulation experiments.

Carbonitrile salts have been previously studied for the treatment of bacterial infections. As reported in a review of the patent literature, Wockhard Ltd. (Aurangabad, India), described a nitrile that displayed inhibitory activity equivalent to Avibactam. Additionally, it exhibited synergy with meropenem against class D ESBL producing strains of *Acinetobacter baumannii* [15]. Additionally, scientists had reported a carbonitrile that displayed features of a broad spectrum metallo-β-lactamase inhibitor [16]. Perhaps not surprisingly, the putative CTX-M-15-inhibitor (ID: MCULE-1352214421-0-56) proposed herein was found to be a carbonitrile.

## 3. Materials and Methods

### 3.1. Examination of the Binding Site by CASTp3.0

The three-dimensional structure of the interacting spot of ‘avibactam’ (used as a reference ligand) located on the bacterial CTX-M-15 was examined using ‘CASTp3.0’ of Tian et al. [22]. It is a tool that provides detailed information regarding the interaction site for a given protein–ligand complex. It utilizes the ‘α-shape method’ for identifying protein features [22]. The ‘α-shape method’ is one of the basic ingredients of computational geometry applied in the aforementioned program [22]. Three-dimensional alpha-shapes were described by Edelsbrunner and Mucke [28]. In the context of protein–ligand binding studies, an alpha-shape is used to represent the protein surface, and ‘discrete triangles flow’ is applied for pocket detection. Such methods rely on the actual geometric information from the protein structure and do not rely on other information, like the grid size and searching directions [29]. The PDB ID 4HBU (Resolution: 1.10 Å) was used as the ‘reference complex’.

### 3.2. Structure Based Virtual Screening

‘MCULE online drug discovery platform’ was employed for screening [30]. It has a special place among other related platforms, as apart from providing modeling tools, it provides facility to quickly order the purchase of the screened out drug candidate [30,31]. The ‘InChIKey’ of ‘avibactam’ was retrieved from the PubChem database and then brought to ‘property calculator’ of the MCULE platform. The values of the acquired ‘avibactam property table’ were then employed to construct the minima and maxima of the ‘screening input parameters’. The values for ‘maxima’ and ‘minima’ generated in the aforementioned manner were, in turn, utilized in the ‘MCULE workflow builder’ [30]. A total of 5,000,000 test ligands were screened against the ‘avibactam binding site’ of the bacterial CTX-M-15 protein. Briefly, ‘the allowed number of Lipinski rule-of-five (RO5) violations’ was entered as 1 in the MCULE workflow builder. This was done to impart due flexibility for the initial filters. The designated maximum number of rotatable bonds was 6. The mass range used was 235.24–295.24 Da. The ‘polar surface area’ range was 118.62–158.62 Å2. The minimum and maximum number of hydrogen bond acceptors was entered as 5 and 10, respectively. The selected maximum number of hydrogen bond donors was four. The value for ‘sampler size’ was provided as 1000, while the similarity cut off was kept at 0.7. The value entered for ‘the maximum number of compounds after sphere exclusion’ was 3,000,000. Molecular descriptors were analyzed by ‘Open Babel Linear Fingerprint’. The remaining options were left untouched and kept as default.

#### Molecular Docking

A processed file (ligands and water molecules removed) was prepared from the PDB ID 4HBU and saved separately. This file was supplied to the MCULE screening platform to be used by AutoDock Vina [24]. A grid of 60 Å × 60 Å × 60 Å^3^ was prepared on the protein. The employed values corresponding to x, y, and z position coordinates required for docking were −6.643882, −2.634176, and 11.573353, respectively. These position coordinates were extracted from PDB ID 4HBU by Discovery Studio Visualizer 4.1.

Further, the docking results (notably for the docked complex of the top screened out inhibitor with the bacterial CTX-M-15 protein) were re-confirmed by two other platforms, namely “YASARA GLOBAL DOCKING” (included within licensed YASARA STRUCTURE) and licensed “DOCKINGSERVER” [Virtua Drug Ltd., Hungary]. It is imperative that YASARA GLOBAL DOCKING retained the essential water molecules in the docking protocol. YASARA DOCKING was based on VINA while docking performed by ‘DockingServer’ was based on Autodock. A Supplementary file related to docking and including detailed docking methodology as implemented in ‘dockingserver’ is provided in Supplementary Text S2. Briefly, docking calculations were carried out using ‘DockingServer’ [32]. A semi-flexibile docking approach was used. The ligand was flexible while the protein was treated as a rigid molecule. The MMFF94 force field was used for energy minimization of the ligand. Gasteiger partial charges were added to the ligand atoms. Non-polar hydrogen atoms were merged, and rotatable bonds were defined. DockingServer used a grid of 20 Å × 20 Å × 20 Å^3^. Docking simulations were performed using Lamarckian genetic algorithm and the Solis and Wets local search method [33]. Detailed information regarding docking parameters, like number of runs, energy evaluations, translational step, quaternion, and torsion steps, is provided in the Supplementary file Supplementary Text S3.

### 3.3. VINA Ranking and ADME-Analyses

All of the test ligands were ranked by VINA [24]. Hence, ligands exhibiting upper VINA ranks were identified and analyzed by SWISS ADME [25] for generation of pharmaco-kinetic profiles. A range of tests, like the Lipinski, Ghose, Veber, Egan, PAINS, and Muegge filter tests, were performed for these ligands. The aforementioned tests examined the drug-likeness of the candidate ligands [26,34,35,36,37]. The ligands from the upper layer that passed a minimum of four of the six aforementioned filters were pinpointed.

### 3.4. Docking Score, ‘Zero RO5 Violation’, ‘Synthetic Accessibility Score’ and TOX-CHECK

The ligands that either exhibited a ‘VINA-docking score’ greater than −6.9 or displayed one or more RO5 violations were rejected. Subsequently, the ligands that displayed a ‘Synthetic accessibility score’ cut-off of greater than 3.0 were also excluded. The remainder were evaluated with the ‘toxicity checker’ function of MCULE [30]. Some structural portion of a ligand might cause toxicity. Certain substructural motifs are known to be found quite often in toxic molecules. Hence, it is sensible to reject test molecules having such motifs. This tool is grounded on over 100 toxic scaffolds. An alert is displayed when a test ligand fails the toxicity test in MCULE [30].

### 3.5. Binding Interactions of the Complex Involving the ‘Top CTX-M-15 Inhibitor’ & ‘Molecular Overlay’

The residues responsible for important binding contacts and other important interactions concerning the complex involving the ‘top CTX-M-15 inhibitor’ were identified by Discovery Studio Visualizer 4.1 and YASARA STRUCTURE version 20.12.24.W.64 [38]. Furthermore, to compare this complex with the reference (CTX-M-15-avibactam complex), a ‘molecular overlay’ diagram was generated by Discovery Studio Visualizer.

### 3.6. Molecular Dynamics Simulation (102.25 Nanoseconds)

The molecular dynamics simulation for the complex consisting of the top CTX-M-15 inhibitor (identified by virtual screening) bound to the bacterial CTX-M-15 enzyme was run with YASARA STRUCTURE version 20.12.24.W.64 in three replicas [38]. The MD simulation setup included an optimization of the hydrogen bonding network [39] to increase the solute stability and a pKa prediction to fine-tune the protonation states of protein residues at the chosen pH of 7.4. NaCl ions were added with a physiological concentration of 0.9%, with an excess of either Na or Cl to neutralize the cell. Energy-minimization was performed to remove bumps and correct the covalent geometry of the structure in YASARA structure version 20.12.24.W.64. After removal of conformational stress by a short steepest descent minimization, the procedure continued by simulated annealing (timestep 2 fs, atom velocities scaled down by 0.9 every 10th step) until convergence was reached, i.e., the energy improved by less than 0.05 kJ/mol per atom during 200 steps. After applying steepest descent and simulated annealing minimizations to remove clashes, the simulation was run for 102.25 nanoseconds using the AMBER14 force field [40] for the solute, GAFF2 [41] and AM1BCC [42] for ligand, and TIP3P for water. The cut-off was 8 Å for Van der Waals forces (the default used by AMBER). No cut-off was applied to electrostatic forces, and the ‘Particle Mesh Ewald algorithm’ was employed. The equations of motions were integrated with a multiple time step of 2.5 fs for bonded interactions and 5.0 fs for non-bonded interactions at a temperature of 298K and a pressure of 1 atm (NPT ensemble was employed) based on certain algorithms that had been defined in a previous article [43]. It is important to mention that the entire simulation protocol (also including the initial energy minimization steps) is taken care of by ‘macros’ in YASARA structure. A graphical user interface is provided to run the simulation through relevant macros. The ‘macro’ identified as ‘md_runfast.mcr’ was used herein to assure smooth execution of the simulation commands. Trajectory analyses were performed by employing the macro ‘md_analyze.mcr’. The macro(s) contained YASARA commands. YANACONDA macro language was used for complex tasks. Publication quality figures including RMSD and RMSF plots were generated by YASARA STRUCTURE version 20.12.24.W.64. Snapshots were taken after every 250 ps. A total of 410 snapshots were captured. Finally, two short video clips of ‘41 s’ and ‘1 min and 22 s’ were recorded.

## 4. Conclusions

The present article describes the in silico binding interactions of bacterial CTX-M-15 enzyme with a new ligand, selected out of a high throughput virtual screen. This ligand, namely 5-Amino-1-(2H-[1,2,4]triazino[5,6-b]indol-3-yl)-1H-pyrazole-4-carbonitrile, satisfied ADMET features and displayed encouraging ‘simulation results’. Advanced plots obtained by trajectory analyses indicated the stability of the proposed protein–ligand complex.

Hence, 5-Amino-1-(2H-[1,2,4]triazino[5,6-b]indol-3-yl)-1H-pyrazole-4-carbonitrile could emerge as a promising seed molecule for CTX-M-15-inhibitor design. Based on these in silico studies, wet laboratory validation is warranted.

## Figures and Tables

**Table 1:** Pharmacokinetic profiling of the four upper ranking putative inhibitors of bacterial CTXM-15 enzyme using the SWISS ADME program.

Features	MCULE- 8226995017-0-1	MCULE- 1352214421-0-56	MCULE- 4732388337-0-31	MCULE- 2070524301-0-1
IUPAC Name	2-(4-Morpholinyl) -4,7-dioxo-1,4,5,6,7,8- hexahydropyrido [2,3-d]pyrimidine -5-carboxylic acid	5-Amino-1-(2H- [1,2,4]triazino [5,6-b]indol-3-yl) -1H-pyrazole -4-carbonitrile	(5S)-7-Amino -2,4-dioxo-5-phenyl -1,3,4,5-tetrahydro -2H-pyrano[2,3-d] pyrimidine-6-carbonitrile	(7R)-2-Amino-7 -(3-nitrophenyl)-6,7 -dihydro[1,2,4] triazolo[1,5-a]pyrimidin-5(1H)-one
Chemical Formula	C_12_H_14_N_4_O_5_	C_13_H_8_N_8_	C_14_H_10_N_4_O_3_	C_11_H_10_N_6_O_3_
Molecular Weight	294.26	276.26	282.25	274.24
XLOGP3	−2.49	1.31	0.62	0.33
RO5 violations	0	0	0	0
H-bond acceptors	6	5	4	5
H-bond donors	3	2	3	2
Rotatable bonds	2	1	1	2
Toplogical PSA (Å²)	124.62	122.09	124.76	131.65
Molar Refractivity	77.26	75.43	73.11	74.13
GI Absorption	Low	High	High	High
BBB-permeation	No	No	No	No
Log S (Ali)	0.42	−3.47	−2.81	−2.66
Lipinski-filter	Yes; 0 violation	Yes; 0 violation	Yes; 0 violation	Yes; 0 violation
Ghose-filter	No; 1 violation: WLOGP < −0.4	Yes	Yes	Yes
Veber-filter	Yes	Yes	Yes	Yes
Egan-filter	Yes	Yes	Yes	No; 1 violation: TPSA > 131.6
Muegge-filter	No; 1 violation: XLOGP3 < −2	Yes	Yes	Yes
PAINS-filter	0 alert	0 alert	0 alert	0 alert
Brenk-filter	0 alert	0 alert	0 alert	1 alert: nitro group
Lead-likeness	Yes	Yes	Yes	Yes
Synthetic accessibility	3.36	2.81	3.65	2.93

## Data Availability

Data is contained within the article or supplementary material.

## Supplementary Materials

The following are available online at https://www.mdpi.com/article/10.3390/antibiotics10050474/s1, Figure S1: A ray-traced picture of the simulated system, Supplementary Text S2: GLOBAL DOCKING RESULT ANALYSIS [YASARA log file], Supplementary Text S3: Methodology used by ‘Docking Server’.
