# Supplementary material for: High Throughput Virtual Screening and Molecular Dynamics Simulation for Identifying a Putative Inhibitor of Bacterial CTX-M-15
Source: Antibiotics (Basel). 2021 Apr 21;10(5):474. doi: 10.3390/antibiotics10050474 (PMC8143117; doi:10.3390/antibiotics10050474)
Supplement: Supplementary file 1 [file antibiotics-10-00474-s001.zip › antibiotics-1195967-supplementary.pdf]

Supplementary File:

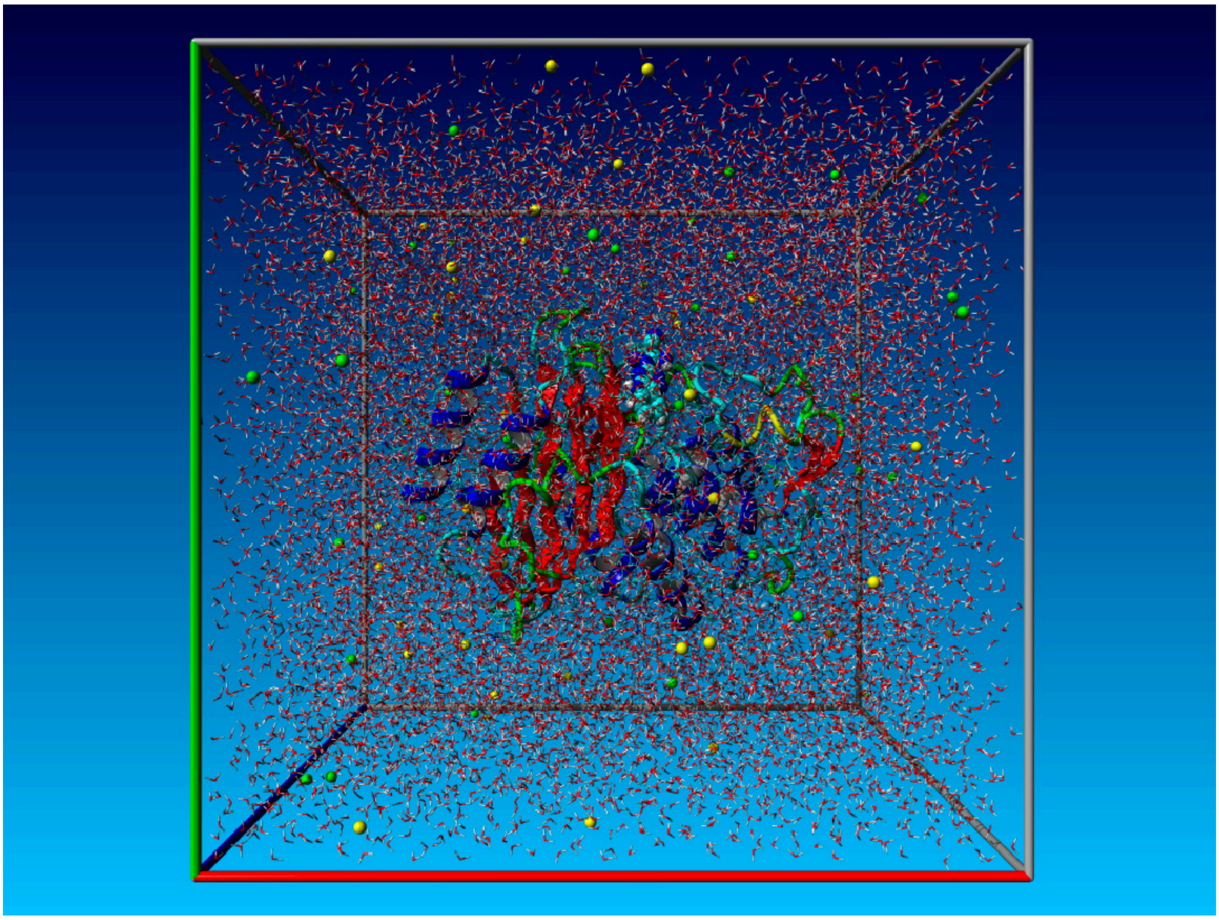

Figure S1: A ray-traced picture of the simulated system.

Supplementary text S2:

Global docking result analysis [YASARA log file]

==============================

25 VINA docking runs of the ligand to the receptor yielded the following results,

sorted by binding energy [more positive energies indicate stronger binding, and negative energies mean no binding]

Run |Bind.energy[kcal/mol]|Dissoc. constant [pM]| Contacting receptor residues

----+---------------------+---------------------+-----------------------------

001 | 000008.0940 | 00000001166861.5000 | A SER 70 A LYS 73 A ASN 104 A TYR 105 A SER 130 A ASN 132 A PRO 167 A THR 168 A ASN 170 A THR 171 A SER 237 A GLY 238 A GLY 239

002 | 000008.0940 | 00000001166861.5000 | A SER 70 A LYS 73 A ASN 104 A TYR 105 A SER 130 A ASN 132 A PRO 167 A THR 168 A ASN 170 A THR 171 A SER 237 A GLY 238 A GLY 239

003 | 000008.0040 | 00000001358282.8750 | A CYS 69 A SER 70 A ASN 104 A SER 130 A ASN 132 A PRO 167 A THR 168 A ASN 170 A THR 171 A LYS 234 A GLY 236 A SER 237 A GLY 238 A GLY 239

004 | 000008.0040 | 00000001358282.8750 | A CYS 69 A SER 70 A ASN 104 A SER 130 A ASN 132 A PRO 167 A THR 168 A ASN 170 A THR 171 A LYS 234 A GLY 236 A SER 237 A GLY 238 A GLY 239

005 | 000007.6640 | 00000002411090.5000 | A SER 70 A LYS 73 A ASN 104 A TYR 105 A SER 130 A ASN 132 A PRO 167 A ASN 170 A THR 171 A THR 216 A SER 237 A GLY 238 A GLY 239

006 | 000007.6640 | 00000002411090.5000 | A SER 70 A LYS 73 A ASN 104 A TYR 105 A SER 130 A ASN 132 A PRO 167 A ASN 170 A THR 171 A THR 216 A SER 237 A GLY 238 A GLY 239

007 | 000007.5340 | 00000003002650.5000 | A SER 70 A LYS 73 A ASN 104 A TYR 105 A SER 130 A ASN 132 A PRO 167 A ASN 170 A THR 171 A SER 237 A GLY 238 A GLY 239

008 | 000007.5340 | 00000003002650.5000 | A SER 70 A LYS 73 A ASN 104 A TYR 105 A SER 130 A ASN 132 A PRO 167 A ASN 170 A THR 171 A SER 237 A GLY 238 A GLY 239

009 | 000007.4520 | 00000003448351.7500 | A SER 70 A LYS 73 A ASN 104 A TYR 105 A TYR 129 A SER 130 A ASN 132 A PRO 167 A THR 168 A ASN 170 A THR 171 A THR 216 A SER 237 A GLY 238 A GLY 239

010 | 000007.4520 | 00000003448351.7500 | A SER 70 A LYS 73 A ASN 104 A TYR 105 A TYR 129 A SER 130 A ASN 132 A PRO 167 A THR 168 A ASN 170 A THR 171 A THR 216 A SER 237 A GLY 238 A GLY 239

011 | 000007.3570 | 00000004048065.2500 | A LEU 155 A GLY 156 A ASP 157 A GLU 158 A THR 159 A ALA 185 A GLN 188 A THR 189 A ARG 191 A ASN 192 A GLY 196 A LYS 197

012 | 000007.3420 | 00000004151859.5000 | A CYS 69 A SER 70 A ASN 104 A SER 130 A ASN 132 A PRO 167 A THR 168 A ASN 170 A THR 171 A LYS 234 A GLY 236 A SER 237 A GLY 238 A GLY 239

013 | 000007.3100 | 00000004382268.0000 | A CYS 69 A SER 70 A ASN 104 A SER 130 A ASN 132 A PRO 167 A ASN 170 A LYS 234 A THR 235 A GLY 236 A SER 237 A GLY 238 A GLY 239

014 | 000007.2420 | 00000004915227.5000 | A SER 70 A ASN 104 A TYR 105 A TYR 129 A SER 130 A ASN 132 A ASN 170 A THR 215 A THR 216 A LYS 234 A GLY 236 A SER 237

015 | 000007.2060 | 00000005223143.5000 | A SER 70 A LYS 73 A ASN 104 A TYR 105 A SER 130 A ASN 132 A GLU 166 A ASN 170 A SER 237 A GLY 238 A GLY 239 A GLY 241 A PRO 268 A LYS 269 A ALA 270 A GLU 271

016 | 000007.2010 | 00000005267408.5000 | A ASP 63 A LEU 155 A GLY 156 A ASP 157 A GLU 158 A THR 159 A SER 182 A ARG 184 A ALA 185 A GLN 188 A THR 189 A ASN 192

017 | 000007.1300 | 00000005938007.0000 | A CYS 69 A SER 70 A LYS 73 A ASN 104 A TYR 105 A SER 130 A ASN 132 A PRO 167 A THR 168 A ASN 170 A THR 171 A SER 237 A GLY 238 A GLY 239

018 | 000007.1260 | 00000005978231.5000 | A GLY 43 A ARG 44 A ARG 65 A PHE 66 A PRO 174 A GLY 175 A PRO 177 A THR 180 A THR 264 A GLN 265 A PRO 266

019 | 000007.1050 | 00000006193925.0000 | A SER 70 A ASN 104 A TYR 105 A TYR 129 A SER 130 A ASN 132 A PRO 167 A ASN 170 A THR 216 A LYS 234 A THR 235 A GLY 236 A SER 237 A GLY 238 A GLY 239

020 | 000007.0200 | 00000007149436.0000 | A SER 70 A ASN 104 A TYR 105 A SER 130 A ASN 132 A ASN 170 A THR 215 A THR 216 A ALA 219 A LYS 234 A GLY 236 A SER 237 A ARG 274

021 | 000007.0010 | 00000007382423.5000 | A SER 70 A LYS 73 A ASN 104 A TYR 105 A SER 130 A ASN 132 A ASN 170 A SER 237 A GLY 238 A GLY 239 A TYR 240 A GLY 241 A PRO 268 A LYS 269 A ALA 270

022 | 000007.0010 | 00000007382423.5000 | A ASP 63 A GLY 156 A ASP 157 A GLU 158 A THR 159 A PHE 160 A SER 182 A ARG 184 A ALA 185 A GLN 188 A THR 189

023 | 000006.9650 | 00000007844898.0000 | A ALA 79 A LYS 82 A LYS 83 A SER 86 A GLU 87 A VAL 142 A SER 147 A VAL 148 A PHE 151

024 | 000006.9500 | 00000008046045.0000 | A SER 70 A LYS 73 A ASN 104 A TYR 105 A SER 130 A ASN 132 A THR 216 A ALA 218 A THR 235 A ARG 274

025 | 000006.7510 | 00000011257744.0000 | A ASP 63 A GLY 156 A ASP 157 A GLU 158 A THR 159 A SER 182 A ARG 184 A ALA 185 A GLN 188 A THR 189 A ASN 192

After clustering the 25 runs, the following 9 distinct complex conformations were found:

Clu |Bind.energy[kcal/mol]|Dissoc. constant [pM]| Contacting receptor residues

----+---------------------+---------------------+-----------------------------

001 | 000008.0940 | 00000001166861.5000 | A SER 70 A LYS 73 A ASN 104 A TYR 105 A SER 130 A ASN 132 A PRO 167 A THR 168 A ASN 170 A THR 171 A SER 237 A GLY 238 A GLY 239

002 | 000008.0040 | 00000001358282.8750 | A CYS 69 A SER 70 A ASN 104 A SER 130 A ASN 132 A PRO 167 A THR 168 A ASN 170 A THR 171 A LYS 234 A GLY 236 A SER 237 A GLY 238 A GLY 239

003 | 000007.3570 | 00000004048065.2500 | A LEU 155 A GLY 156 A ASP 157 A GLU 158 A THR 159 A ALA 185 A GLN 188 A THR 189 A ARG 191 A ASN 192 A GLY 196 A LYS 197

004 | 000007.2420 | 00000004915227.5000 | A SER 70 A ASN 104 A TYR 105 A TYR 129 A SER 130 A ASN 132 A ASN 170 A THR 215 A THR 216 A LYS 234 A GLY 236 A SER 237

005 | 000007.2010 | 00000005267408.5000 | A ASP 63 A LEU 155 A GLY 156 A ASP 157 A GLU 158 A THR 159 A SER 182 A ARG 184 A ALA 185 A GLN 188 A THR 189 A ASN 192

006 | 000007.1260 | 00000005978231.5000 | A GLY 43 A ARG 44 A ARG 65 A PHE 66 A PRO 174 A GLY 175 A PRO 177 A THR 180 A THR 264 A GLN 265 A PRO 266

007 | 000006.9650 | 00000007844898.0000 | A ALA 79 A LYS 82 A LYS 83 A SER 86 A GLU 87 A VAL 142 A SER 147 A VAL 148 A PHE 151

008 | 000006.9500 | 00000008046045.0000 | A SER 70 A LYS 73 A ASN 104 A TYR 105 A SER 130 A ASN 132 A THR 216 A ALA 218 A THR 235 A ARG 274

009 | 000006.7510 | 00000011257744.0000 | A ASP 63 A GLY 156 A ASP 157 A GLU 158 A THR 159 A SER 182 A ARG 184 A ALA 185 A GLN 188 A THR 189 A ASN 192

While the table above lists the best binding energy in each cluster, it is sometimes

helpful to also look at the energy spread [average and standard deviation], the

dissociation constant has been recalculated from the average binding energy:

Clu |Members|Bind.energy spread [kcal/mol]|Dissoc. constant [pM]

----+-------+-----------------------------+---------------------

001 | 005 | 000007.4372+-000000.3851 | 00000003535841.6913

002 | 006 | 000007.4427+-000000.3015 | 00000003503367.8404

003 | 001 | 000007.3570+-000000.0000 | 00000004048367.3411

004 | 002 | 000007.1310+-000000.1110 | 00000005928420.4022

005 | 002 | 000007.1010+-000000.1000 | 00000006236330.6297

006 | 001 | 000007.1260+-000000.0000 | 00000005978663.4065

007 | 001 | 000006.9650+-000000.0000 | 00000007845448.8655

008 | 001 | 000006.9500+-000000.0000 | 00000008046613.2435

009 | 001 | 000006.7510+-000000.0000 | 00000011258514.7405

Point charges and dihedral barriers were obtained from the AMBER03 force field.

Supplementary text S3:

Methodology used by DockingServer:

My Proteins | My Ligands | My Dockings

ligand056 to 4hbu - HYDROLASE / HYDROLASE INHIBITOR

Geometry | Energy | 2D plot | Interaction Table | HBPlot | Methods | Gallery | Param

Computational Methods

Docking calculations were carried out using DockingServer (*Bikadi, Hazai, 2009*). The MMFF94 force field (*Halgren, 1998*) was used for energy minimization of ligand molecule (***ligand056***) using DockingServer. Gasteiger partial charges were added to the ligand atoms. Non-polar hydrogen atoms were merged, and rotatable bonds were defined.
Docking calculations were carried out on ***4hbu - HYDROLASE / HYDROLASE INHIBITOR*** protein model. Essential hydrogen atoms, Kollman united atom type charges, and solvation parameters were added with the aid of AutoDock tools (*Morris, Goodsell et al., 1998*). Affinity (grid) maps of 20×20×20 Å grid points and 0.375 Å spacing were generated using the Autogrid program (*Morris, Goodsell et al., 1998*). AutoDock parameter set- and distance-dependent dielectric functions were used in the calculation of the van der Waals and the electrostatic terms, respectively.
Docking simulations were performed using the Lamarckian genetic algorithm (LGA) and the Solis & Wets local search method (*Solis and Wets, 1981*). Initial position, orientation, and torsions of the ligand molecules were set randomly. Each docking experiment was derived from 100 different runs that were set to terminate after a maximum of 2500000 energy evaluations. The population size was set to 150. During the search, a translational step of 0.2 Å, and quaternion and torsion steps of 5 were applied.

References

Bikadi, Z., Hazai, E.
Application of the PM6 semi-empirical method to modeling proteins enhances docking accuracy of AutoDock
J. Cheminf. **1**, 15 (2009)

T. A. Halgren
Merck molecular force field. I. Basis, form, scope, parametrization, and performance of MMFF94
Journal of Computational Chemistry **17 (5-6)**, 490-519 (1998)

G. M. Morris, D. S. Goodsell, et al.
Automated docking using a Lamarckian genetic algorithm and an empirical binding free energy function
Journal of Computational Chemistry **19 (14)**, 1639-1662 (1998)

F. J. Solis and R. J. B. Wets
Minimization by Random Search Techniques
Mathematics of Operations Research **6 (1)**, 19-30 (1981)

##Note: The ligand having ID as MCULE-1352214421-0-56 has been referred as 'ligand056' for brevity in the screenshot shown above.

Docking Parameters

Advanced Settings

tstep	0.2
qstep	5.0
dstep	5.0
rmstol	2.0
ga_pop_size	150
ga_num_evals	2500000
ga_num_generations	540000
ga_run	100
